# Evidence in clinical reasoning: a computational linguistics analysis of 789,712 medical case summaries 1983–2012

**DOI:** 10.1186/s12911-015-0136-8

**Published:** 2015-03-21

**Authors:** Bastian M Seidel, Steven Campbell, Erica Bell

**Affiliations:** Wicking Dementia Education and Research Centre, University of Tasmania, Hobart, TAS 7001 Australia; School of Health Sciences, University of Tasmania, Launceston, TAS 7250 Australia

**Keywords:** Clinical reasoning, Medical intuition, Evidence-based practice

## Abstract

**Background:**

Better understanding of clinical reasoning could reduce diagnostic error linked to 8% of adverse medical events and 30% of malpractice cases. To a greater extent than the evidence-based movement, the clinical reasoning literature asserts the importance of practitioner intuition—unconscious elements of diagnostic reasoning. The study aimed to analyse the content of case report summaries in ways that explored the importance of an evidence concept, not only in relation to research literature but also intuition.

**Methods:**

The study sample comprised all 789,712 abstracts in English for case reports contained in the database PUBMED for the period 1 January 1983 to 31 December 2012. It was hypothesised that, if evidence and intuition concepts were viewed by these clinical authors as essential to understanding their case reports, they would be more likely to be found in the abstracts. Computational linguistics software was used in 1) concept mapping of 21,631,481 instances of 201 concepts, and 2) specific concept analyses examining 200 paired co-occurrences for ‘evidence’ and research ‘literature’ concepts.

**Results:**

‘Evidence’ is a fundamentally patient-centred, intuitive concept linked to less common concepts about underlying processes, suspected disease mechanisms and diagnostic hunches. In contrast, the use of research literature in clinical reasoning is linked to more common reasoning concepts about specific knowledge and descriptions or presenting features of cases. ‘Literature’ is by far the most dominant concept, increasing in relevance since 2003, with an overall relevance of 13% versus 5% for ‘evidence’ which has remained static.

**Conclusions:**

The fact that the least present types of reasoning concepts relate to diagnostic hunches to do with underlying processes, such as what is suspected, raises questions about whether intuitive practitioner evidence-making, found in a constellation of dynamic, process concepts, has become less important. The study adds support to the existing corpus of research on clinical reasoning, by suggesting that intuition involves a complex constellation of concepts important to how the construct of evidence is understood. The list of concepts the study generated offers a basis for reflection on the nature of evidence in diagnostic reasoning and the importance of intuition to that reasoning.

## Background

The 21^st^ Century evidence-based medicine movement has placed less emphasis on intuition and tacit knowing in forming sound clinical judgements [1,2]. Yet a growing debate about the evidence-practice divide in medicine over the last decade asserts the limited value of research for complex practitioner decision-making [3-6]. It has been argued that practitioner judgement must be better valued because guidelines synthesised from the ‘gold standard’ evidence of clinical trials, reviews and meta-analyses are about groups, not individuals [6,7]. Such evidence has been described as suffering from an insufficiency linked to the narrowness of its aims versus the breadth of clinical judgement required for ‘point of care’ complexities [3,8].

This tension between scientific ‘evidence-based’ thinking and intuitive thinking is suggested also by the clinical reasoning literature. Yet this emerging body of literature offers little clear consensus on what is clinical reasoning [9]. Since 1983 (the period encompassed by this study) over a thousand papers are listed in PUBMED as journal articles with the term ‘clinical reasoning’ in the abstract or title. Strategies for improving clinical reasoning processes refer to building knowledge acquisition, data gathering, data processing, as well as metacognition capacities that manage bias through self-awareness [10-12]. Practice setting—a construct that includes the interactions between patient, practitioner, environment and other ambient contextual factors—has also been argued to be important for clinical reasoning [9,13]. A machine-driven ‘cognitive mapping methodology’ has been used to represent the multidimensional, non-linear, dynamic nature of clinical reasoning to confirm the importance of a sound knowledge base, as well as hypothesis generation and problem representation mechanisms. The clinical encounter was found to activate a series of cognitive actions: tapping of clinical knowledge reservoirs, mobilising and enriching of scripts and accessing of bio-psycho-social knowledge, parallel control of these processes by metacognition [14]. Yet whatever model is used to describe clinical reasoning, such models do not fit current diagnostic ‘evidence-based’ clinical practice guidelines [15,16].

Better understandings of clinical reasoning could help manage diagnostic error. Diagnostic error has been linked to 8% of adverse medical events and 30% of malpractice cases [17]. Diagnostic error has been mostly (75%) found comprise of cognitive issues that are about how information is collected, integrated and verified [17]. Yet while a recent review found a hundred papers suggesting interventions to decrease the likelihood of cognitive-based errors in diagnostic reasoning, those few that had been tested involved trainees in artificial contexts removed from practice [18].

Bias, associated with non-analytical reasoning, has been described in one review as neutralised by reflective reasoning that is particularly important for diagnostic accuracy in complex cases [19,20]. Emotional intelligence and empathy in sound clinical reasoning for quality medical diagnoses are less well valued than the role of hypothetico-deductive cognition [21,22]. Yet emotional intelligence has been elsewhere demonstrated to be the only variable that contributes to a clinical reasoning construct [23]. Clinical ‘first impressions’ have also been found to deliver similar diagnostic reasoning performance as directed structured analytic thinking [24]. Further, a 2010 review found very little evidence to support claims for diagnostic errors being associated with non-analytical reasoning [25]. Fast, unconscious, contextual process (System 1) thinking is no more associated with bias or diagnostic error than slow, analytical and conscious (System 2) thinking—encouraging both can reduce error [26].

Alternative theories to resolve such tensions include the supposedly analytical and intuitive elements of clinical decision-making in ‘balanced’ models [27,28]. These may include a ‘distributed intelligence’ approach in which patients and practitioners share problem-solving in the practice setting [29]. For example, ‘cognitive continuum theory’ asserts that clinical reasoning can be better described on an analysis-intuition continuum such that much reasoning may be ‘quasi-rational’ involving elements of both [30]. Such balanced theories appear supported by evidence of both analytic and nonanalytic elements found in clinical reasoning processes using functional magnetic resonance imagining and ‘think aloud protocols’ [31].

The language in which practitioner case reports are summarised offers elements of ‘real world’ case scenarios. Such scenarios have been the foundation of teaching clinical reasoning: hypothesis generation, pattern recognition, formulation of context, diagnostic test interpretation, differential diagnosis as well as diagnostic verification [32]. We aimed to use machine-driven techniques for quantifying large qualitative datasets to address the question of how clinical practitioners conceptualise evidence in one kind of case scenario genre—abstracts of case reports—particularly as it relates to understanding the role of intuition versus research literature.

## Methods

The description of the method that follows draws upon the method described in our previous Bayesian-based machine-driven studies of other large language databases [33].

### Research question

The research question was: ‘How important is a concept of evidence in published summaries of clinical cases? What other concepts do practitioners associate with an evidence concept in their case summaries, particularly concepts to do with research literature and intuition?’

### Study sample

The study design involved treating abstracts of case reports as indicative evidence of what authors of those reports selected as important to understanding them. It was assumed that, if evidence and literature concepts were viewed by these clinical authors as essential to understanding their case reports, they would be more likely to be found in the abstracts of those reports. The study sample comprised all abstracts in English for case reports contained in the database PUBMED for the period 1 January 1983 to 31 December 2012. Case reports are defined in the PUBMED database as ‘Clinical presentations that may be followed by evaluative studies that eventually lead to a diagnosis’. This provided a total of 789,712 distinct abstracts all of which were included in the study. At approximately three abstracts per page, this corpus equates to approximately 263,237 pages or approximately 877 books, assuming 250 pages per book.

Abstracts were grouped in five-year sub periods specified in Table 1, which provides the frequency of abstracts.

**Table 1:** **Numbers of abstracts in the sample, by period of analysis**

**Year**	**Frequency**
2008-2012	180,482
2003-2007	165,788
1998-2002	138,967
1993-1997	129,107
1988-1992	120,917
1983-1987	54,451
Total	789,712

### Analytic procedure

The analytic approach involved the application of a machine-driven, computational linguistics approach. This entailed content analysis of concepts in the abstracts using the software Leximancer v4.0 (Leximancer, Brisbane, Australia ). This Bayesian-based software has been extensively applied in hundreds of studies across different disciplines, including in health [33-41]. Many public domain software tools listed in digital libraries such as http://dirt.projectbamboo.org/ lack validation studies. Across four criteria—cost, usability, as well as published validation studies and applications—Leximancer compares favourably with a wide range of other competing commercial text analytics products described in business intelligence product assessments [42].

A text block of about a paragraph in size is the unit of analysis in Leximancer. Each paragraph may contain one or more concepts. Leximancer ‘learns’ from an uploaded language dataset to create a network of such concepts. The key output of the Leximancer software is a concept map with supporting data. The concept map provides a visual representation of all found and/or user-selected concepts in a corpus, based on their proximity or overall co-occurrence. In summary, concept mapping in Leximancer can be described as an iterative numerical model that simulates relationships between concepts to produce a complex network system with associated visualisations of data [43]. Further detailed discussion of algorithms and other technical aspects of the software are given in a published validity study [43].

The analysis involved two standardised stages to quantify the conceptual content of the abstracts using the software (Leximancer version 4), as follows.

#### Stage 1: Concept mapping

This stage obtained a conceptual overview of the case report abstracts. The software produced a concept map with supporting frequency and co-occurrence statistics for all concepts mapped from the abstracts. In this study, the automated features of the software were used to derive the concept map. Very similar concepts were merged either automatically by the software or by the researchers. Thus the concept of ‘evidence’ is likely to include the concept of ‘evidence-based’. However, the concept ‘literature’ will not necessarily include the concept ‘review’ though the two may often be semantically proximate, something the method was designed to also assess. Concepts that did not contribute to an understanding of the content of the abstracts were not used as mapping concepts. That is, ideationally void concept words such as ‘appropriate’ were not used as mapping concepts. Terms such as ‘aims’, ‘methods’ or ‘findings’, which form structural features of abstracts, were also not used as mapping concepts. This does not mean this content was excised from the analysis, merely that such concept words were not used as organising terms under which the content of the abstracts was grouped. Accordingly, in Stage 1 the concept map was used to display all found concepts and their semantic proximity to one another across the entire database of 789,712 abstracts. In this stage, the software mapped 21,631,481 instances of 201 concepts found in 4,808,728 text blocks.

#### Stage 2: Evidence concept analysis

This stage involved identification and extraction of data for two key concepts: the concepts of ‘evidence’ and ‘literature’. In this stage, 59,341 instances of the evidence concept were analysed to identify concepts that were most to least frequently paired with the evidence concept. Accordingly, 200 types of paired co-occurrences for the evidence concept were extracted and examined. The data providing frequencies and likelihood of paired co-occurrences were obtained. Overall likelihoods of the evidence concept occurring for each sub period of the study were also obtained. The 167,737 instances of the literature concept were similarly analysed.

Manual scanning was also performed to do checks of the validity of the data analyses. The multiple data viewing windows in Leximancer facilitated these checks. That is, Leximancer allows the analyst to see all text blocks for a concept and also extract each instance of a concept to view it in the original data file. These checks were performed to help to ensure that the text blocks selected by Leximancer as part of a concept definition of, for example, ‘evidence’, did in fact always include the word ‘evidence’ or a term associated with it semantically. It is estimated that a total of 5% of the corpus or approximately 44 books were manually scanned for this purpose.

### Strengths and limitations of the method

Our method offers indicative evidence of how medical practitioners, engaged in the evidence-making fora of journals, conceptualise evidence when summarising the most important aspects of a case report. It does not offer conclusive evidence of how they use evidence in practice or even in the detail of case reports. Rather, the study suggests how they conceptualise that evidence when summarising clinical cases in the genre ‘case report abstracts’. This is a critical distinction. Further, the study is not indicative of all case reports written for journals, only of those with structured abstracts. Only 60.1% of all 1,312,960 case reports for the period 1983–2012 listed in PUBMED have abstracts used in this study.

Leximancer, like other such machine-based data mining tools, offers scoping of large datasets but not fine-grained explanations of their conceptual nuances. Accordingly, the study was designed to offer broad, not nuanced, findings about how practitioners publishing case reports conceptualise evidence in summaries of those reports i.e. typical semantic features. It is useful to questioning broad assumptions about the nature of those concepts and their presence over time.

Strengths of the study include the novelty of both the database and the method. The study sample as a whole represents 48% of the total 1,638,946 case reports in the PUBMED database for all years (i.e. not just for the study period) and all languages where an abstract in English exists—a substantial corpus. The PUBMED database of case report abstracts has not previously been analysed for this purpose using data mining methods, although the field of computational ‘text analytics’ offers many opportunities for such investigations.

### Ethics statement

The study is a content analysis of published material in peer reviewed journals. No data were collected from human subjects.

## Results

### Conceptual overview

**Figure 1:**
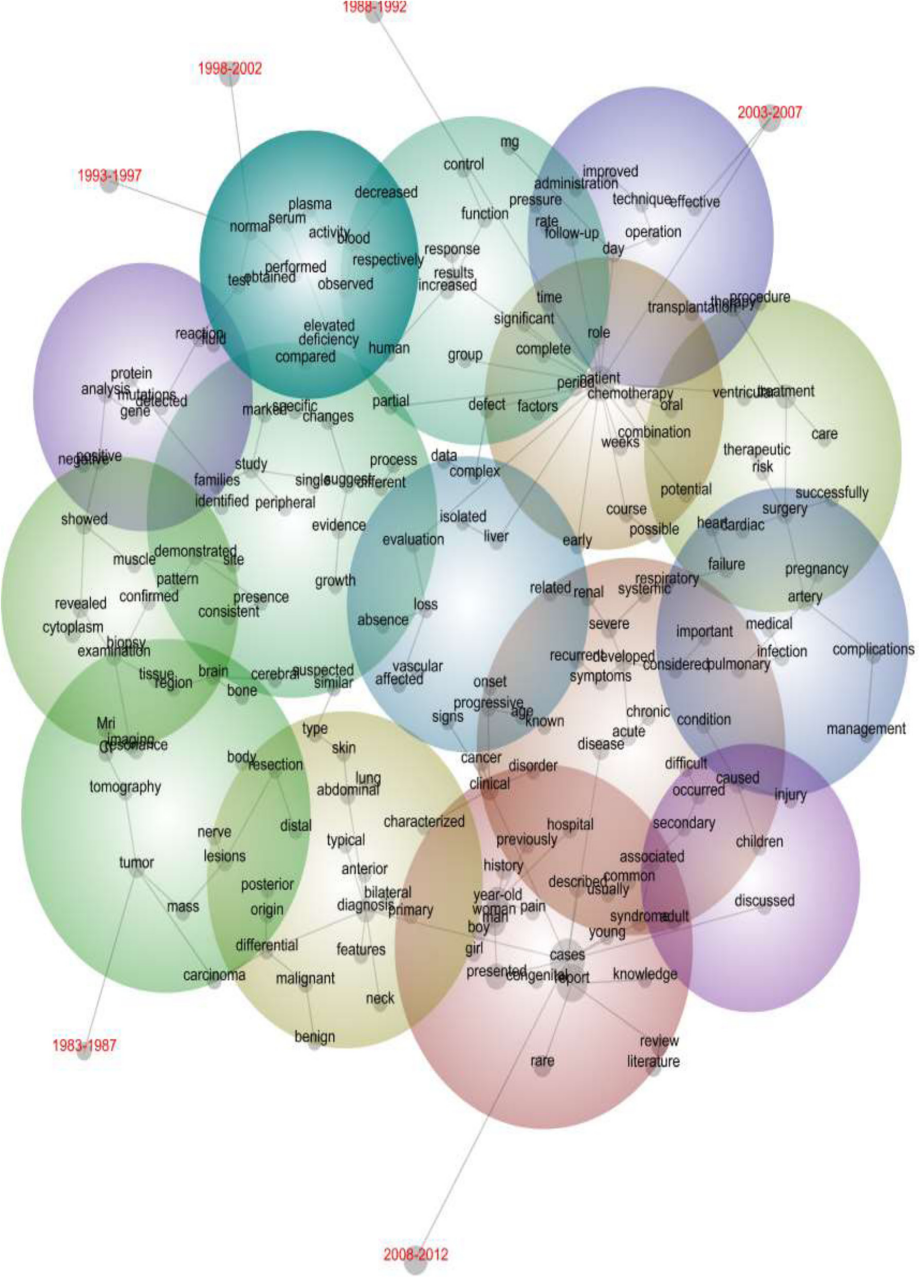
**Concept map of all 789,712 medical case summaries.**

The concept map in Figure 1 offers a spatial representation of all instances of the 201 concepts found across the 789,712 abstracts in this study. There were 21,631,481 instances of these concepts in 4,808,728 text blocks. The concept map is colour coded according to the traditional colour wheel with redder or ‘warmer’ spheres being where the more frequent concepts are likely to be found and bluer spheres being where the least frequent concepts are more likely to be found. Concepts that are proximate on the map have greater semantic similarity and tend to occur together, although the placement of a concept is determined by overall co-occurrences. The size of the grey dots indicates the extent to which a concept co-occurs with all other concepts. The grey lines suggest typical semantic connections across multiple concepts i.e. not simply paired co-occurrences. The map is designed to offer a bird’s-eye view of the data, useful to data scoping prior to more detailed analysis.

Figure 1 suggests that the evidence concept is located in semantic proximity to less common concepts, being placed in one of the darker green spheres. The evidence concept is connected to one typical storyline: growth → evidence → suggest. Through the concept ‘suggest’ the evidence storyline branches out like the spokes of a wheel to:

- →different → process
- →changes → specific
- →single → study.

This suggests that evidence as a concept is not only attached to empirical studies but also to dynamic concepts to do with growth, difference and processes.

In contrast, the literature concept is part of another kind of typical storyline: literature → review → report → cases. Through report and cases, the literature storyline branches out (again, like the spokes of a wheel but not including the semantically far concepts) to:

- →knowledge
- →young → syndrome
- →described
- →presented
- →congenital
- →rare

This indicates that the research literature has tended to be constructed in terms of literature reviews closely aligned to the way in which case reports themselves are conceptualised—as might be expected if literature reviews are commonly used to justify the unusualness of a case. Literature is linked to specific syndromes or particular knowledge and descriptions, making sense of presentations, and congenital and rare disease.

Notably, the two concepts of evidence and literature are not semantically similar i.e. they are not proximate to one another. In summary, evidence is linked to amorphous clinical reasoning storylines like process through the amorphous concept of suggest—a term linked to diagnostic hunches. In contrast, literature → review is not linked to such amorphous clinical reasoning concepts. This suggests clinical reasoning involving research literature is possibly narrower and more descriptive than clinical reasoning involving evidence which is dynamic and involves process understandings.

### Overall concept relevance

Table 2 provides the detail of instances of all 201 concepts. The relevance of a concept relates to the percentage frequency of text blocks in which a concept is found, relative to the frequency of the most common concept ‘case’. Within the cells, concepts are ordered by descending fractional percentages. Thus Table 2 shows that the evidence concept has a relevance of only 5%. Evidence, as such, does not appear to be a very important concept in these summaries of case reports. In contrast, the literature concept has a relevance of 13%. Literature is in fact much more relevant than evidence. This raises the question of whether clinical case reasoning involving literature and what can be known through literature is far more dominant than clinical case reasoning involving evidence and complexity in these data.

**Table 2:** **Ranked Concept List (all concepts)**

**Concept**	**Relevance**
cases	100%
report	80%
patient	77%
treatment	35%
presented	34%
year-old	33%
woman; man; diagnosis	30%
clinical	22%
rare	22%
disease	19%
tumor	18%
results; surgery; associated; syndrome	17%
cytoplasm; described	16%
caused	15%
review; artery; showed; developed	14%
therapy; lesions; severe; literature	13%
study	12%
revealed; infection; acute; performed; complications; examination	11%
abdominal	10%
imaging; normal; symptoms; resonance; carcinoma; suggest	9%
Ct; occurred; systemic; children; increased; pain; discussed	8%
effective; pulmonary; previously; renal; tissue; demonstrated; day; features; malignant; bone; possible; type; positive; common; recurrent; improved; successfully; mass; observed	7%
blood; chronic; primary; condition; history; management; complete; hospital; function; transplantation; confirmed; liver; cancer; analysis; biopsy; weeks; related; considered; detected; age; course; resection; time; tomography	6%
MRI, important; evaluation; failure; gene; cardiac; mutations; progressive; changes; chemotherapy; lung; factors; procedure; significant; combination; differential; skin; injury; operation; loss; ventricular; muscle; nerve; response; identified; early; test; pregnancy; technique; serum; defect; bilateral; evidence	5%
region; disorder; signs; activity; risk; brain; affected; presence; follow-up; potential; anterior; neck; families; period; origin; different; heart; medical; secondary; usually; cerebral; congenital; consistent; elevated; control; decreased; adult; posterior; reaction; body	4%
Mg; isolated; site; negative; deficiency; vascular; characterized; benign; peripheral; oral; knowledge; partial; similar; care; human; onset; therapeutic; boy; pattern; fluid; specific; rate; known; role; typical; girl; respiratory; pressure; process; young; marked; complex; growth; difficult; obtained; administration; distal; protein; data	3%
group; plasma; absence; compared; suspected; respectively; single	2%

However, Table 2 also suggests that clinical cases involve a wide number of what might be described as reasoning concepts with the most common including: diagnosis (32%); associated (17%); described (16%); caused (15%); review (14%); showed (14%); revealed (11%). The least common reasoning concepts are: absence, compared, and suspected, which all have a 2% relevance, as well as concepts such as characterized, knowledge, similar, pattern, known, typical, process, marked, complex, growth, data, which all have a 3% relevance. This suggests that summaries of case reports rely explicitly on broad brush diagnostic judgements of what can be associated or described or understood causally or reviewed or showed or revealed. They tend not to rely explicitly on concepts to do with what is suspected or what can be compared or what may be absent or what may be about diagnostic complexity, patterns and underlying processes.

### The literature concept

Table 3 provides the list of paired co-occurrences for the literature concept in all 200 of the other concepts. For example, the likelihood percentage for the concept of review shows the extent to which all text blocks with the concept review also contain the literature concept i.e. so the likelihood percentages do not of course add up to 100%. That is, the most likely term to be paired with literature is the term review. Not surprisingly, based on the concept map in Figure 1, Table 3 suggests that the top seven concepts paired with literature also include discussing, knowledge and data concepts (with knowledge and data being previously noted as one of the least common concepts) and also, of course, the case concept. The next eight common paired co-occurrences for the literature concept (ranging from 12%-9% likelihood) also include two of the previously noted least common reasoning concepts: similar and compared. This suggests further that literature is linked with not only broad practitioner research concepts, but also more superficial reasoning concepts such as discussed, what is known previously, what is similar or rare, what can be described or compared or observed. These concepts might not be categorised as ‘deep’ reasoning concepts that address underlying processes, but rather presenting features of cases.

**Table 3:** **Paired co-occurrences for the literature concept**

**Concept**	**Likelihood**
review	86%
discussed	17%
knowledge	16%
report	14%
data; medical; cases	13%
previously	12%
similar; management; described	10%
Rare; therapeutic; compared; features	9%
presented; clinical	8%
related; benign; adult; condition; malignant; carcinoma; care; injury	7%
primary; associated; differential; different; possible; known; role; young; identified; important; risk; complications; children; common; pregnancy; study; tumor	6%
congenital; diagnosis; potential; origin; secondary; syndrome; disease; neck; suggest; factors; occurred; usually; treatment; history; process; group; cancer; surgery; disorder; type; single; isolated; course; infection; evaluation; oral; age; systemic	5%
evidence; lesions; body; girl; vascular; difficult; specific; considered; renal; typical; patient; caused; period; site; follow-up; early; combination; results; bilateral; pattern; acute; boy; nerve; procedure; successfully; bone; affected; chronic; posterior; characterized; technique; complex; absence; recurrent; observed; developed; rate; significant; respectively; year-old; symptoms; therapy; woman; man; time; human; signs; effective; operation; pulmonary; respiratory; cytoplasm	4%
liver; region; tissue; presence; skin; artery; resection; cerebral; imaging; severe; onset; resonance; distal; cardiac; transplantation; abdominal; failure; changes; lung; obtained; analysis; families; chemotherapy; reaction; complete; brain; performed; anterior; confirmed; mass; growth; response; consistent; pain; progressive; partial; muscle; examination; revealed; defect; heart; loss; demonstrated; increased; hospital; suspected; function; peripheral; fluid; administration	3%
MRI, CT, deficiency; biopsy; improved; weeks; tomography; control; test; positive; pressure; detected; ventricular; negative; normal; activity; blood; marked; showed; plasma; elevated; day	2%
decreased; serum; protein; mutations; mg; gene	1%

The least common eight paired co-occurrences for the literature concept or those ranging from 2-1% are more narrow biomedical and clinical concepts such as ‘serum’ (1%) and ‘tissue’ (2%). It appears therefore that the more a concept relates to the broader concepts of clinical management the more likely it is to be linked to the literature concept. The more a concept relates to the biomedical and clinical detail of patient management, the less likely it is to be found with the literature concept. Overall, the distribution of concepts in terms of the likelihood percentages suggests that most of these clinical concepts are not very likely to be found with the literature concept. Scrutiny of the placement of reasoning concepts such as ‘detected’ (2%) and ‘suspected’ (3%) further confirms that diagnostic reasoning concepts to do with underlying processes are not found with the literature concept.

Examination of the likelihood figures by sub period of the analysis suggests that there is a difference by sub period in the likelihood of the literature concept being found. That is, references to literature have not remained static with a 4% likelihood overall of being found in any one text block over the years 2003–2012 and a 3% likelihood over the years 1983–2002. That is, it appears that summaries of case reports are increasingly using the literature concept.

### The evidence concept

Table 4 similarly provides the paired co-occurrences for the evidence concept in all 200 other concepts. It suggests that there is no single concept that is overwhelmingly likely to be associated with the evidence concept. If Figure 1 suggested that the evidence concept is a complex and amorphous concept in case diagnosis, Table 4 supports this by suggesting that evidence in these abstracts involves a very wide array of concepts with a 6-1% likelihood range. The evidence concept is most likely to be found paired with concepts to do with recurrent events or patterns (6%) and follow up (5%). It is as likely to be found with a nebulous concept such as ‘absence’ (4%) as it is with ‘presence’ (4%). It is as likely to be found with the concept of ‘data’ (3%) as it is with the concept of ‘signs’ (3%) that may be associated with medical hunches. The concepts least likely to be associated with evidence, or those with a 1% likelihood, tend to be broad health, medical and service setting concepts such as procedure or management or hospital or technique. The concept of literature itself has only a 2% likelihood of being associated with the evidence concept. The likelihood of the evidence concept being found has remained static at 1% from 1983–2012. Therefore, it appears that the concept of evidence has not become more important in more recent years.

**Table 4:** **Paired co-occurrences for the evidence concept**

**Concept**	**Likelihood**
recurrent	6%
follow-up	5%
suggest; absence; showed; presence	4%
MRI; CT; biopsy; role; data; disease; marked; demonstrated; process; elevated; clinical; human; revealed; activity; obtained; brain; peripheral; examination; consistent; growth; normal; changes; malignant; infection; systemic; function; cytoplasm; study; response; similar; negative; progressive; test; signs; specific; serum; protein; factors; analysis; chemotherapy; period; bone; pattern; families; significant; plasma; vascular; origin; fluid; single; increased; related; time; primary; cerebral; liver	3%
gene; evaluation; typical; tumor; observed; group; tomography; previously; complete; confirmed; history; identified; reaction; tissue; positive; lesions; resonance; region; disorder; possible; suspected; detected; differential; body; site; nerve; abdominal; known; considered; imaging; muscle; respectively; effective; chronic; potential; renal; patient; control; developed; loss; early; deficiency; features; acute; transplantation; onset; lung; knowledge; mutations; affected; failure; different; blood; cardiac; results; compared; course; heart; injury; resection; medical; improved; complex; type; weeks; pulmonary; mass; symptoms; decreased; risk; carcinoma; presented; isolated; pressure; bilateral; associated; ventricular; therapy; severe; condition; rate; young; skin; age; cancer; distal; important; performed; respiratory; characterized; care; therapeutic; defect; combination; administration; partial; occurred; children; adult; day; oral; diagnosis; described; surgery; cases; syndrome; year-old; secondary; operation; common; report; neck; review; caused; pain; congenital; benign; woman; literature; treatment; man; girl; mg; anterior; posterior; usually	2%
boy; artery; pregnancy; discussed; successfully; procedure; management; hospital; rare; difficult; complications; technique	1%

## Discussion

The background section suggested that clinical reasoning literature does not support simplistic mistrust of practitioner intuition or the more unconscious elements of diagnostic reasoning. It suggests that intuitive ‘System 1’ thinking has a role as important as rational ‘System 2’ thinking that includes explicit use of research [26].

This study suggests that evidence is a fundamentally patient-centred, intuitive concept in summaries of what is important to case reports. Evidence is far more linked to amorphous, dynamic concepts suggestive of suspected underlying processes and diagnostic hunches. Its typical storylines across multiple concepts include, but are not limited to, research studies. The evidence concept is as likely to be found with scientific concepts such as ‘data’ as it is with concepts such as ‘signs’ that may be associated with medical intuitions.

The study also suggested that, at least in abstracts, the use of research literature in clinical reasoning is linked to more superficial reasoning concepts or specific knowledge and descriptions or presenting features of cases. The more a concept relates to the broader concepts of clinical management, the more likely it is to be linked to the literature concept (itself most often found in the concept review i.e. as in literature review). The more a concept relates to the biomedical and clinical detail of patient management, the less likely it is to be found with the literature concept.

The study suggests that the use of research literature in diagnostic reasoning may have become more prevalent, in contrast to intuitive practitioner evidence-making, at least in these data. Of course, such a finding may be an artefact not simply of changes in the practice of clinical reasoning but rather in the requirements of journals in reporting cases. Literature is by far the most dominant concept with an overall relevance of 13% versus 5% for evidence. Further, literature has been increasing in relevance since 2003 while the concept of evidence has remained static.

These findings should be interpreted in light of the fact that clinical case summaries include a wide range of reasoning concepts. The fact that the most frequent reasoning concepts are concepts to do with what can be associated or described or understood causally and so on raises questions about the role of the literature concept in a constellation of more superficial reasoning concepts. The fact that the least present types of reasoning concepts relate to diagnostic hunches to do with underlying processes, such as what is suspected, also raises questions about whether intuitive practitioner decision-making, found in a constellation of dynamic, process concepts, has become less important.

## Conclusions

These findings raise more questions than the method can answer. Clearly, the research literature is a critical part of clinical reasoning, but how is it used in practice in relation to more intuitive aspects of clinical reasoning? This study has suggested that literature, primarily literature reviews often used to establish the unusualness of a case, is selected by clinical authors of case reports as important to summary representations of those cases but also that intuition may have a critical role. Future work applying this method should involve examining relationships between intuition and literature reviews, as well as other kinds of evidence, in different kinds of informal unstructured texts gained from clinical reasoning situations. In the meantime, this study adds support to the existing corpus of research on clinical reasoning, by suggesting that intuition involves a complex constellation of concepts important to how the construct of evidence is understood. The list of concepts the study generated offers a basis for teachers, students and clinicians to reflect on the nature of evidence in diagnostic reasoning and the importance of intuition to that reasoning.
